# Choroidal and retinal displacements after vitrectomy with internal limiting membrane peeling in eyes with idiopathic macular hole

**DOI:** 10.1038/s41598-019-54106-0

**Published:** 2019-11-26

**Authors:** Kensuke Goto, Takeshi Iwase, Tomohiko Akahori, Kentaro Yamamoto, Eimei Ra, Hiroko Terasaki

**Affiliations:** 1 Department of Ophthalmology, Nagoya University Graduate School of Medicine, Nagoya, Japan; 2 Department of Ophthalmology, Akita University Graduate School of Medicine, Akita, Japan

**Keywords:** Medical imaging, Outcomes research

## Abstract

It has been reported that the macular region of the retina is displaced after vitrectomy with internal limiting membrane (ILM) peeling in eyes with macular hole (MH), but the displacements of the deeper layers of the eye, e.g. RPE and choroid are unclear following the surgery. We used optical coherence tomography (OCT) and OCT angiography (OCTA) to obtain 3 mm × 3 mm *en face* images before, and 2, 4, and 8 weeks following the vitrectomy with internal limiting membrane (ILM) peeling from 22 eyes of 22 patients with a MH. The OCT and OCTA images showed displacements of the fovea and choroidal intermediate vessels postoperatively. The degree of displacement of the choroid was significantly less than that of the retina (*P* < 0.001). The displacements of the choroidal bifurcations were significantly correlated to their preoperative distance from the optic disc (*r* = −0.467, *P* < 0.001) and they were significantly correlated with the retinal displacements (*r* = 0.535, *P* < 0.001). The retina was displaced inferiorly and centripetally, but these localized displacements were not observed in the choroid. In conclusion, clinicians need to be aware of these displacements when evaluating the subfoveal choroid following the surgery because the displacement is different between the retina and the choroid.

## Introduction

Earlier studies have shown that an idiopathic macular hole (MH) can be treated by vitrectomy^1–4^, and the closure rate was significantly increased by peeling the internal limiting membrane (ILM) during the vitrectomy^2–6^. Analyses of pre- and postoperative fundus photographs have shown that the retina is displaced in the macular region following the MH closure by vitrectomy with ILM peeling^7–10^. Displacement of the retina is also caused by vitrectomy with ILM peeling in other retinal diseases^11^.

Optical coherence tomography angiography (OCTA) is noninvasive and safe and has enabled clinicians to investigate the retinal vasculature *in situ* repeatedly in short intervals^12,13^. Akahori *et al*. evaluated the displacements of the retina using the OCTA *en face* images of the superficial retinal capillary plexus (SCP), and they concluded that the retina was displaced nasally, inferiorly, and centripetally in the macular region^10^.

The retinal vessels observed in the fundus photographs are mostly those of the inner retina, and the SCP observed in the OCTA *en face* images is located in the inner retina^10^. This indicated that the macula displacements are displacements of the inner retina. However, the displacements of the deeper layers of the retina, e.g. RPE and choroid following vitrectomy with ILM peeling are unclear.

It has been reported that the thickness of the choroid^14,15^ and its blood flow^16,17^ are altered by retinochoroidal diseases. Other studies have reported that the choroidal thickness is reduced in both the affected and the unaffected contralateral eye compared to that of age- and sex-matched control eyes in eyes with an idiopathic MH^18,19^. In addition, the retinal and choroidal thicknesses in the OCT images were reduced following vitrectomy with ILM peeling for a MH^20^. If the choroid is displaced following vitrectomy with ILM peeling, the displacement would probably affect the postoperative choroidal thickness and blood flow.

The OCT instruments can obtain *en face* images at different retinal depths including the intermediate choroidal plexus (ICP)^12,13^, which can be used to measure the displacements of the choroid following vitrectomy with ILM peeling. The displacements have been measured for the SCP^10^ but not for the ICP.

Thus, the purpose of this study was to determine whether the retina and choroid in the macular region are displaced following the vitrectomy with ILM peeling in eyes with a MH.

## Methods

### Ethics statement

The procedures used in this cross-sectional retrospective study were approved by the Ethics Committee of the Nagoya University Hospital, Nagoya, Japan, and they conformed to the tenets of the Declaration of Helsinki. All of the patients signed an informed consent form before the surgery.

### Subjects

We reviewed the medical records of patients with a unilateral, idiopathic MH who had undergone successful MH closure by vitrectomy at the Nagoya University Hospital from October 2017 to September 2018. Comprehensive ophthalmic examinations were performed on all the patients. In addition, OCTA, fundus autofluorescence (FAF), and SD-OCT (Spectralis^®^, Heidelberg Engineering, Heidelberg, Germany) images were obtained before, and 2, 4, and 8 weeks following the surgery. The exclusion criteria included eyes with severe cataract, macular diseases, history of other ocular diseases, and high myopia of more than −5 diopters (D).

The basal diameter and minimum diameter of the MH was measured using the horizontal SD-OCT images. The size and the location of the ILM peeled areas were determined by the recorded surgery videos. The ratio of the diameters of the ILM peeing area to optic disc area was calculated with the imageJ software. The FAF images were obtained by the Spectralis^®^ instrument with an excitation wavelength of 488 nm and emission wavelength of >500 nm using the high-resolution mode with the automatic real time protocol.

### Optical coherence tomography angiography (OCTA)

**Figure 1:**
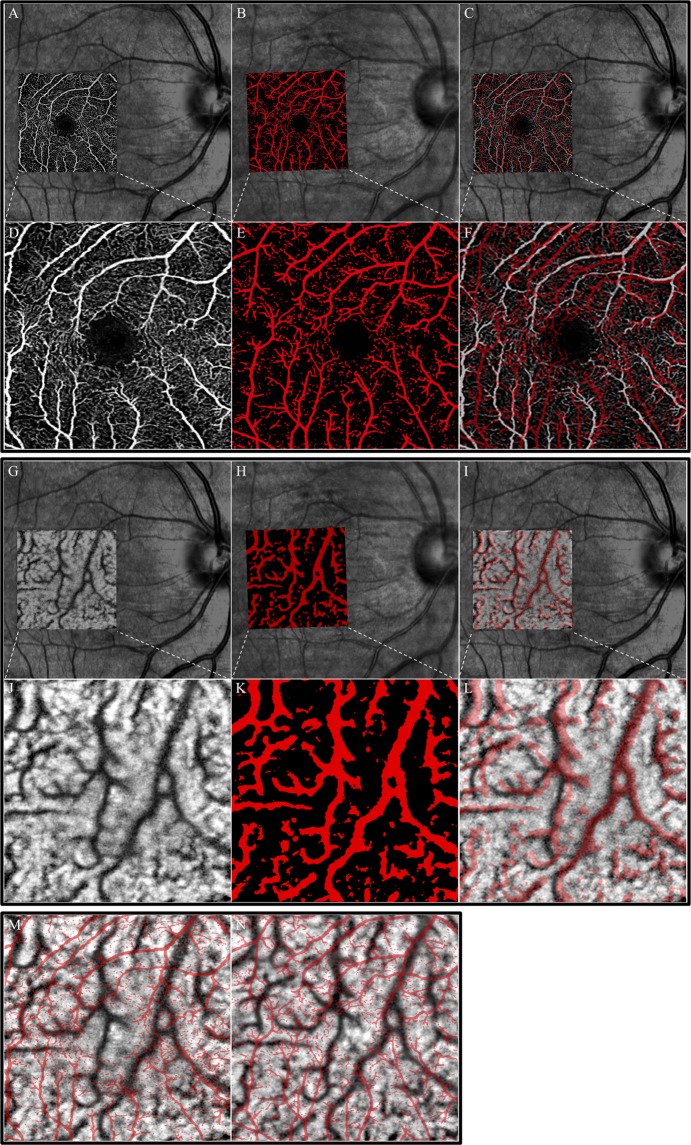
Representative fundus photographs and images of the foveal superficial capillary plexus (SCP) and intermediate choroidal plexus (ICP) determined by optical coherence tomography angiography (OCTA) from a 64-year-old woman with a macula hole. (Upper). Upper panels show fundus photograph and superimposed OCTA image of the SCP **(A–C**) and the lower panels show 3 × 3 mm^2^ *en face* OCTA images of the microvasculature network centered on the fovea. (**D–F**) The left image shows the preoperative images and the retinal vasculature is shown by white lines. (**D**) The middle image shows an OCTA image at 2 weeks after the vitrectomy and the retinal vasculature is shown as red lines. (**E**) The right image shows the superimposed preoperative (white) and postoperative (red) images. (**F**) The preoperative and postoperative images were adjusted by matching retinal vessels around the optic disc. (Middle). Upper panels show fundus photograph and superimposed OCT image of the ICP. (**G**–**I**) The lower panels show 3 × 3 mm^2^ *en face* OCT images of the ICP centered on the fovea. (**J–L**) The left image shows preoperative image and the ICP is shown by black lines. (**J**) The middle image shows fundus photograph with an OCT image at 2 weeks after the vitrectomy and the choroidal vasculature is shown as red lines. (**K**) The right images show the preoperative (black) image superimpose on the postoperative (red) images (**L**). (Bottom) The left image shows the superimposed preoperative retinal (red) and choroidal (black) vasculature images. **(M**) The middle image shows the superimposed postoperative retinal (red) and choroidal (black) vasculature images (**N**).

We used the Angioplex (Zeiss Meditec. Inc, Germany) to obtain the 3 × 3 mm^2^ *en face* images of the microvasculature in the macular region^10^. The images of the SCP that was provided automatically by the embedded software of the OCTA were used to measure the displacements of the retina. The images of the ICP obtained from the same area of the 3 × 3 mm^2^ *en face* images using OCT function were used to measure the displacements of the choroid (Fig. 1). The SCP is located between the ILM and the inner plexiform layer, and the ICP is located between 89 and 115 µm posterior to the RPE layer. The two graders (KG and TI) reviewed all of the images, and excluded low quality OCT angiographic images from the analysis. The vascular displacements were measured independently by these two observers who were masked to the other clinical findings.

### Measurements of displacement using OCTA images

**Figure 2:**
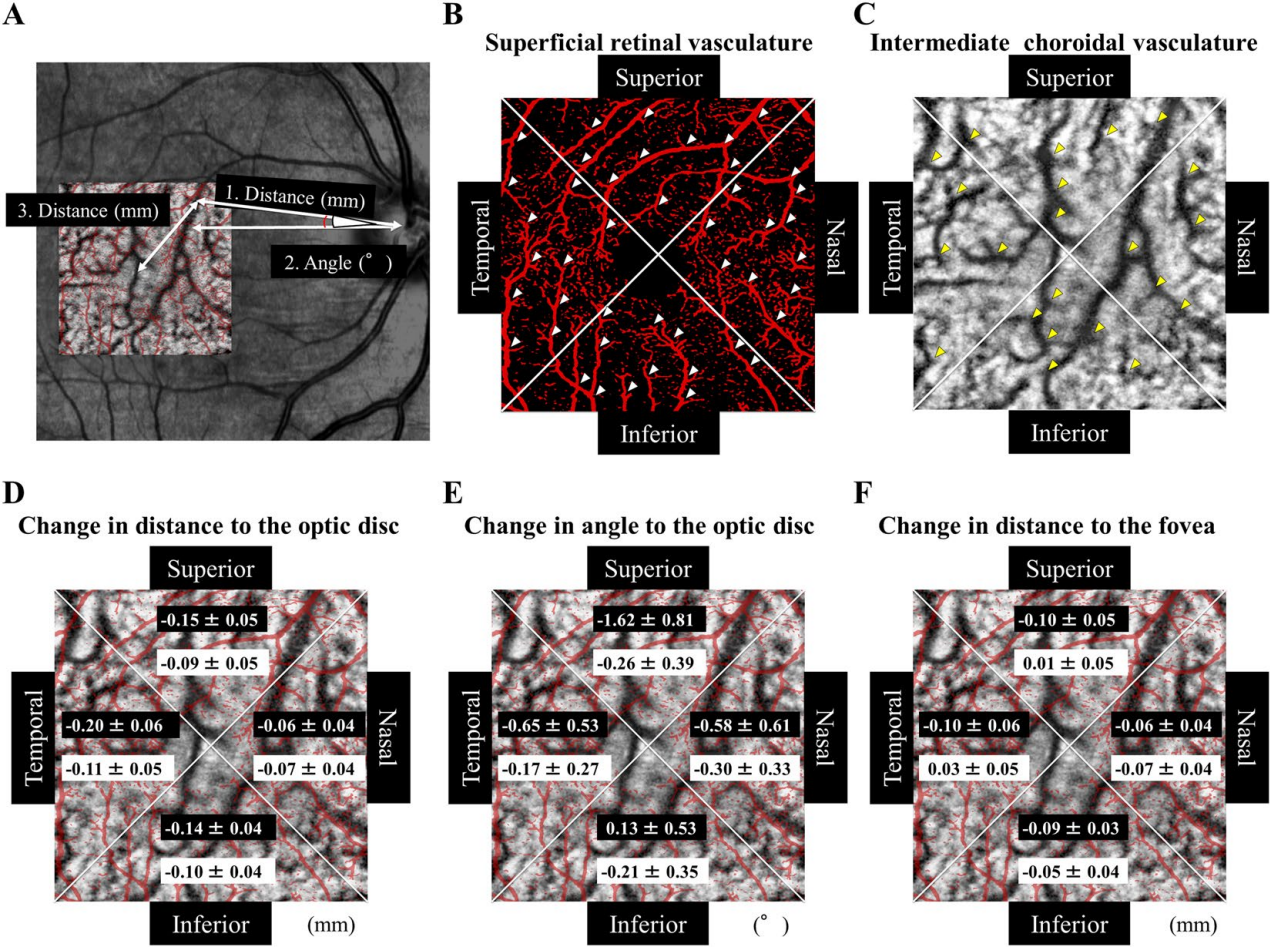
Displacement measurements using optical coherence tomography angiography images and the results for the four quadrants. Displacement measurements using optical coherence tomography angiographic (OCTA) images and the results for the quadrants. (**A**) Plot of distances between the bifurcation and the center of the optic disc (#1), angle formed by a line between the center of the optic disc and the bifurcation point and a horizontal line through the center of the optic disc (#2). The distance between the bifurcation and the fovea (#3). The OCTA images are divided into the four quadrants; the temporal, superior, nasal, and inferior in the retina (**B**) and the choroid. (**C**) Changes in the distance from the optic disc and the retinal and choroidal vessel or bifurcations (**D**), in the angle from the optic disc to the retinal and choroidal vessel bifurcations (**E**), and in distance between the fovea and the retinal and choroidal vessel bifurcations (**F**) between the preoperative and Week 2 in each sector is shown. The retinal displacement is shown in red on a white background and the choroidal displacement is shown in black on the white background.

The displacements of retina were measured using the OCTA and OCT images as described in detail^10^. In brief, we first selected an easily identifiable retinal vessel bifurcation in the SCP image and choroidal vessel bifurcation in the ICP image. The distance between the bifurcation and the center of the optic disc was measured (#1 in Fig. 2A). Then, the angle formed by a line between the center of the optic disc and the bifurcation point and a horizontal line through the center of the optic disc was measured (#2 in Fig. 2). In addition, the distance between the bifurcation and the fovea was measured (#3 in Fig. 2).

### Surgical techniques

We performed a standard three-port PPV with the Alcon Constellation 25-gauge (G) system (Alcon Laboratories, Inc., Fort Worth, TX, USA). Core vitrectomy was performed, and if necessary, a posterior vitreous detachment was created. Then, the ILM was peeled circumferentially from the retina using ILM-peeling forceps in all cases. Then, we performed a peripheral vitrectomy with shaving and inspected carefully the periphery over 360 degrees carefully in all cases. Finally, fluid-air exchange was performed followed by the injection of 20% sulfur hexafluoride (SF_6_) or 12% perfluoropropane (C_3_F_8_) into the vitreous cavity. The patients were instructed to maintain a prone position until a closure of the MH was confirmed with OCT following the surgery.

We performed cataract surgery and implanted a foldable acrylic IOL into the capsular bag on all 17 phakic eyes.

### Statistical analyses

We used one-way analysis of variance (ANOVA) to evaluate the significance of the relationship between the displacements in the four quadrants and the chronological data. We used Pearson’s correlation coefficient test to evaluate the significance of the associations between the changes in the displacement of each bifurcation and the angle formed and the preoperative distance and angle. All statistical analyses were performed using the Statistical Package for Social Sciences for Windows 21.0 (SPSS Inc, Chicago, IL).

## Results

### Demographics of patient

Thirty-eight eyes of 38 patients with a MH underwent the successful MH closure surgery during the period. Sixteen eyes were excluded; 2 for prior vitrectomy, 1 for macular degeneration, 10 for high myopia, and 3 for severe cataract that prevented an acquisition of high quality OCTA images. Finally, 22 eyes of 22 patients (mean age, 66.0 ± 9.3 years) were studied. The demographics of the patients and the surgical procedures are shown in Table 1. The mean size of the ILM peeled area was 3.73 ± 0.55 disc diameters (DD). The mean number of retinal bifurcations that was identified was 50.1 ± 8.3 per eye and the mean number of choroidal bifurcations was 19.8 ± 2.5 per eye.

**Table 1:** Clinical characteristics of subjects.

Characteristics	n = 22
Age (year)	66.0 ± 9.3
Male: female (patients)	7: 15
Right: Left (eyes)	12: 10
Preoperative BCVA (logMAR)	0.60 ± 0.29
Postoperative BCVA (logMAR)	0.27 ± 0.20
Axial length (mm)	24.0 ± 1.5
FAZ size (mm^2^)	0.39 ± 0.10
MH stage 2: 3: 4 (eyes)	6: 9: 7
Basal MH size (μm)	679 ± 330
Minimum MH size (μm)	330 ± 186
PPV + PEA + IOL/PPV (eyes)	17: 5
ILM peeling size (DD)	3.73 ± 0.55
Gas tamponade (C_3_F_8_: SF_6_)	4: 18

MH: macular hole, BCVA: best-corrected visual acuity, logMAR: logarithm of minimum angle of resolution, FAZ: foveal avascular zone, PPV: pars plana vitrectomy, PEA: phacoemulsification and aspiration, IOL: intra-ocular lens, ILM: internal limiting membrane, DD: disc diameter, C_3_F_8_: Octafluoropropane, SF_6_: Sulfur hexafluoride.

### Changes of foveal position

The fovea was displaced following the surgery, and the changes in the average distance between the fovea and the optic disc were shortened by 0.14 ± 0.06 mm and the angle was rotated downwardly by an average of 0.89 ± 0.73°.

### Changes in distance between retinal or choroidal bifurcations and optic disc

**Figure 3:**
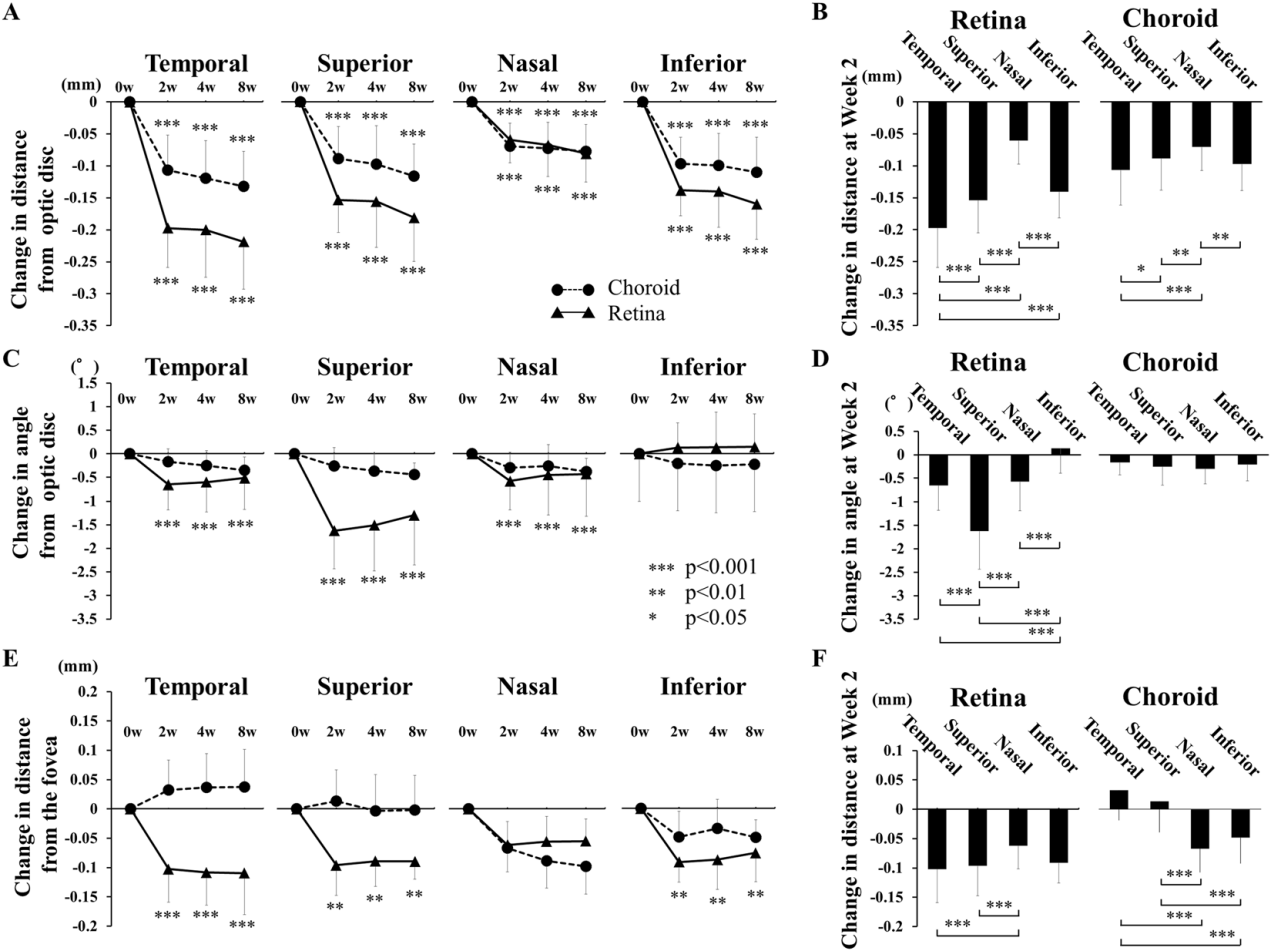
Displacements of the retinal and choroidal bifurcations in the quadrants after surgery and comparisons among the quadrants. (**A**) Distances from the center of the optic disc and retinal and choroidal vessel bifurcations was significantly shorter in all of the quadrants throughout the postoperative period (*P* < 0.001). However, there were no significant differences in the distance for both the retina and choroid after Week 2. The distance in the choroid was significantly shorter than that in the retina in the temporal, superior, and inferior quadrants throughout postoperative period. (**B**) Comparisons of the changes in the distance to the optic disc in the 4 quadrants at Week 2. (**C**) Angle from the optic disc and the retinal vessel bifurcations is rotated significantly downward in the temporal, superior, and inferior quadrants at Week 2 (*P* < 0.01), but the angle in the choroidal vessel bifurcations is not significantly changed after the surgery. (**D**) Comparisons of the changes in the angle in the 4 quadrants at Week 2. (**E**) Distance from the fovea and retinal vessel bifurcations was significantly shorter in the temporal, superior, and inferior quadrants throughout the postoperative period following surgery (*P* < 0.001). (**F**) Comparisons of the changes in the distance to the fovea in the 4 quadrants at Week 2. Each point represents the mean ± SD. *P < 0.05, **P < 0.01, ***P < 0.001 between 2 comparisons.

The mean preoperative distance between the retinal bifurcation and the center of the optic disc was 4.46 mm, and it was significantly reduced to 4.32 mm (*P* < 0.001) at 2 weeks postoperatively. The mean distances were also reduced significantly in all four quadrants, and the reduction was maintained throughout the postoperative period (Table 2, Fig. 3A).

**Table 2:** Changes in distance and angle between bifurcations and the optic disc or the fovea.

		Quadrant	Week 2	Week 4	Week 8	*P*-value
Change in distance from optic disc (mm)	Retina	Temporal	−0.197 ± 0.062	−0.200 ± 0.074	−0.219 ± 0.074	<0.001
		Superior	−0.154 ± 0.051	−0.156 ± 0.072	−0.181 ± 0.069	<0.001
		Nasal	−0.061 ± 0.037	−0.069 ± 0.050	−0.083 ± 0.046	<0.001
		Inferior	−0.139 ± 0.040	−0.141 ± 0.057	−0.161 ± 0.056	<0.001
	Choroid	Temporal	−0.106 ± 0.054	−0.120 ± 0.058	−0.132 ± 0.054	<0.001
		Superior	−0.089 ± 0.050	−0.098 ± 0.061	−0.116 ± 0.050	<0.001
		Nasal	−0.071 ± 0.037	−0.074 ± 0.041	−0.079 ± 0.043	<0.001
		Inferior	−0.097 ± 0.042	−0.100 ± 0.050	−0.111 ± 0.055	<0.001
Change in angle from optic disc (°)	Retina	Temporal	−0.653 ± 0.529	−0.603 ± 0.627	−0.514 ± 0.660	<0.001
		Superior	−1.624 ± 0.812	−1.510 ± 0.970	−1.296 ± 1.059	<0.001
		Nasal	−0.577 ± 0.607	−0.447 ± 0.844	−0.429 ± 0.900	<0.001
		Inferior	0.134 ± 0.526	0.135 ± 0.749	0.145 ± 0.700	0.68
	Choroid	Temporal	−0.166 ± 0.265	−0.249 ± 0.317	−0.347 ± 0.281	0.97
		Superior	−0.258 ± 0.392	−0.364 ± 0.381	−0.443 ± 0.244	0.775
		Nasal	−0.298 ± 0.331	−0.264 ± 0.457	−0.375 ± 0.281	0.995
		Inferior	−0.206 ± 0.352	−0.251 ± 0.251	−0.223 ± 0.351	0.967
Change in distance from fovea (mm)	Retina	Temporal	−0.102 ± 0.057	−0.108 ± 0.056	−0.109 ± 0.071	<0.001
		Superior	−0.096 ± 0.051	−0.090 ± 0.043	−0.090 ± 0.030	0.002
		Nasal	−0.062 ± 0.040	−0.056 ± 0.043	−0.055 ± 0.038	0.114
		Inferior	−0.091 ± 0.034	−0.088 ± 0.050	−0.076 ± 0.049	0.005
	Choroid	Temporal	0.032 ± 0.051	0.036 ± 0.058	0.038 ± 0.064	0.752
		Superior	0.013 ± 0.053	−0.004 ± 0.062	−0.002 ± 0.059	0.847
		Nasal	−0.067 ± 0.041	−0.089 ± 0.046	−0.098 ± 0.047	0.098
		Inferior	−0.049 ± 0.044	−0.034 ± 0.049	−0.049 ± 0.030	0.825

**Figure 4:**
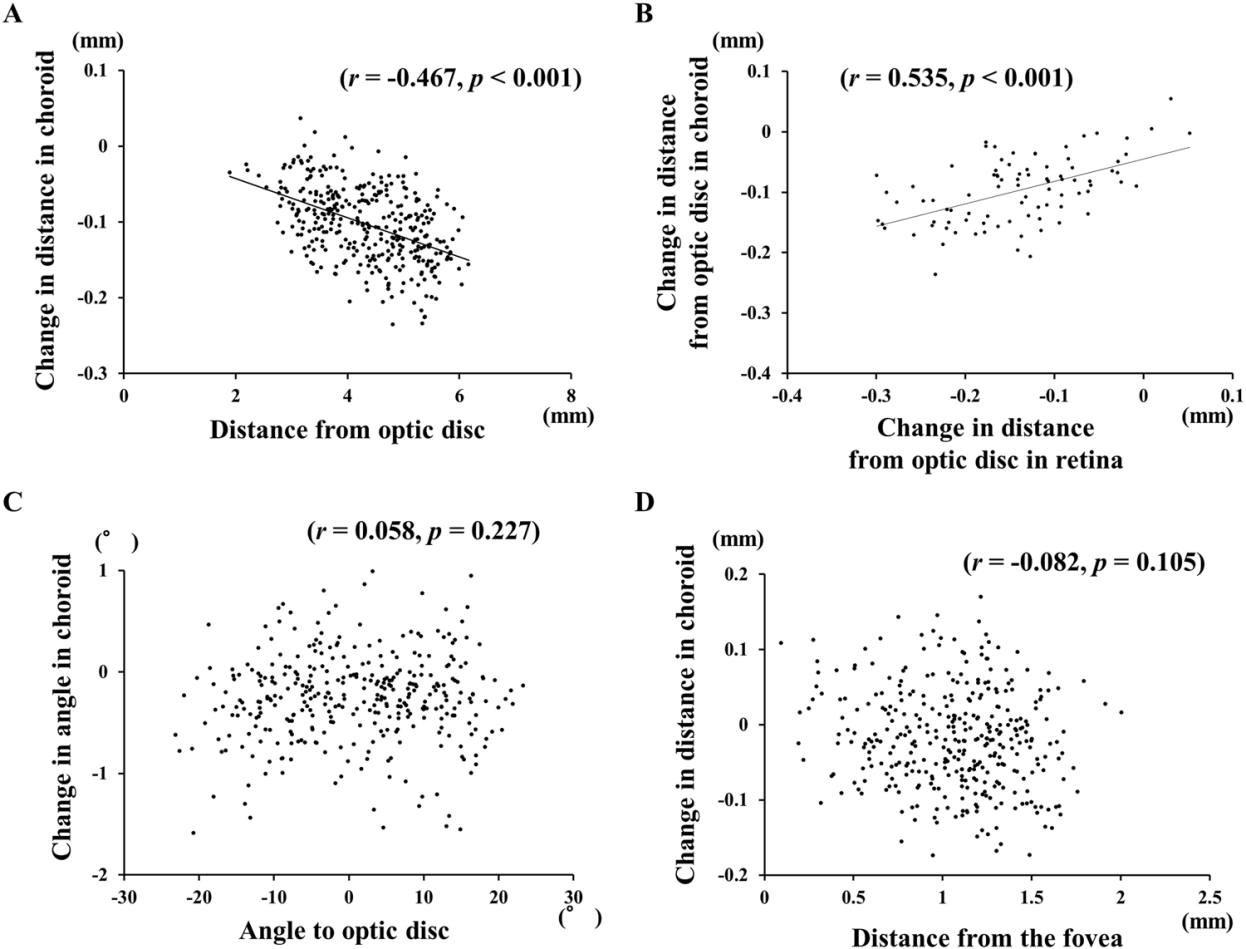
Graphs showing the relationship between the changes in the distance from the optic disc to the choroidal vessel bifurcations, changes in the distance from the optic disc to the retinal and choroidal vessel bifurcations, changes in the angle and the angle from the optic disc to retinal vessel bifurcations, and changes in the distance from the fovea to the choroidal vessel bifurcations. (**A**) Plot of choroidal displacements at 2 weeks following the surgery. The changes in the distances were significantly correlated with the preoperative distance from the optic disc (r = −0.467*, P* < 0.001). (**B**) The changes in distance from the optic disc to the choroidal bifurcations was significantly correlated with that in the retina (r = 0.535, *P* < 0.001). (**C**) Plot of the changes in the angle of the choroid at 2 weeks following surgery. The changes in angle were not significantly correlated with the preoperative angle made by the line through the center of the optic disc to the fovea and the optic disc to the choroidal bifurcations. (**D**) Plot of the choroidal displacements at 2 weeks following surgery. The changes in distance were not significantly correlated with the preoperative distance from the fovea.

The mean preoperative distance between the choroidal bifurcations and the center of the optic disc was 4.40 mm, and it was significantly reduced to 4.30 mm (*P* < 0.001) at 2 weeks postoperatively. The mean distances were also reduced significantly in all four quadrants, and the reductions were maintained throughout the postoperative period. No significant differences were observed in the distances during postoperative periods in the both of retina and choroid in all of the quadrants (Fig. 3B). A significant difference was observed in the distance in the both of retina and choroid among the 4 quadrants at Week 2 (*P* < 0.001; Fig. 3B). The displacement of each choroidal bifurcation at Week 2 was significantly correlated with its preoperative distance from the choroidal bifurcation to the optic disc (*r* = −0.467, *P* < 0.001; Fig. 4A).

### Changes in angle of retinal and choroidal vessel bifurcations

The mean preoperative angle formed by a line between the center of the optic disc and the retinal bifurcation point and a horizontal line through the center of the optic disc was −7.9°, and it was significantly rotated downwardly to −8.6° at 2 weeks following surgery (*P* < 0.001, Fig. 3C). In addition, the angle was significantly rotated downward in the temporal, superior, and nasal quadrants at 2 weeks following surgery (*P* < 0.01). A significant difference was observed in the angle among the 4 quadrants at Week 2 following surgery (*P* < 0.001; Fig. 3D). The changes in the angles were not altered significantly during the postoperative period.

On the other hand, the mean preoperative angle formed by a line between the center of the optic disc and the choroidal bifurcation point and a horizontal line through the center of the optic disc was −7.9°, and it was changed to −8.1° at Week 2 which was not a significant. The changes in the angle at each choroidal bifurcation at Week 2 were not significantly correlated with the preoperative angle to the optic disc (Fig. 4C).

### Changes in distance between retinal and choroidal bifurcations and fovea

The changes in the distance between the fovea and the retinal vessel bifurcations was significantly smaller in the temporal, superior, and inferior quadrant following the surgery (Fig. 3E, Table 2). On the other hand, the distance between the fovea and the choroidal vessel bifurcations was not significantly changed in all of the quadrants following the surgery. However, the distance between the fovea and the bifurcations of the choroidal vessel was in opposite directions between the superior, temporal quadrants and nasal, inferior quadrants (Fig. 3F).

### Relationships between displacements in choroid and other factors

The changes in the distance between the retinal and choroidal bifurcations and the changes in the angle to the optic disc were not significantly correlated with any parameters including the size of the MH, the size of ILM peeled area, the axial length, improvement of BCVA, the type of gas tamponade, surgeon, or the type of surgery using single linear regression analysis. However, when evaluating the change in distance from the optic disc, the degree of the displacement of the quadrants of each eye (22 eyes × 4 quadrants) of the choroid at Week 2 was significantly and positively correlated with that of the retina at Week 2 (*r* = 0.535, *P* < 0.001; Fig. 4B).

### OCT and FAF findings following surgery

**Figure 5:**
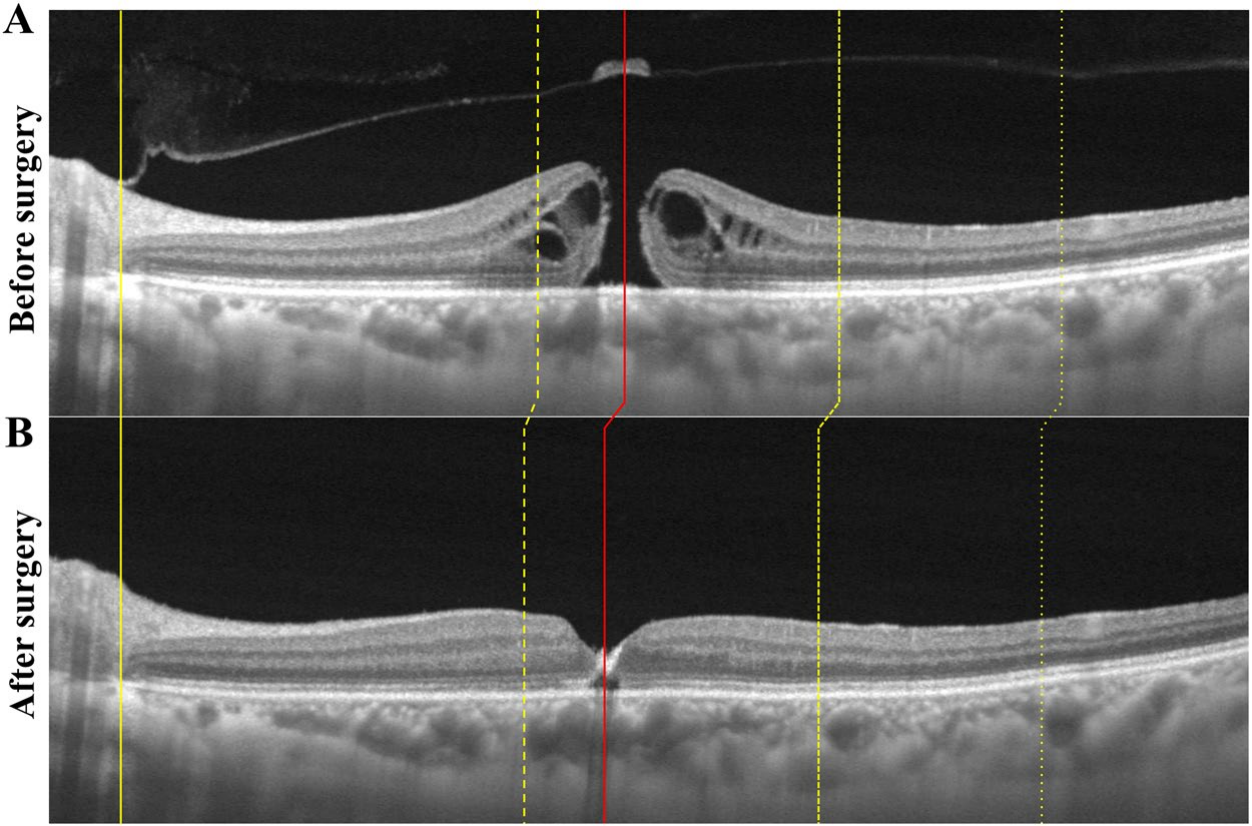
Representative swept source optical coherence tomography (SS-OCT) images of an eye with a macular hole before and 2 weeks after the surgery. (**A**) SS-OCT image of an eye with a macular hole (MH). (**B**) SS-OCT image after the vitrectomy with ILM peeling shows successful closure of the MH and the displacement of the retina and choroid. The yellow line indicates the temporal edge of the optic disc, and no displacement was observed after the surgery. The fovea was displaced after the surgery (red line) and the choroidal vessels were also displaced after the surgery (yellow dot line) especially in the temporal quadrants.

**Figure 6:**
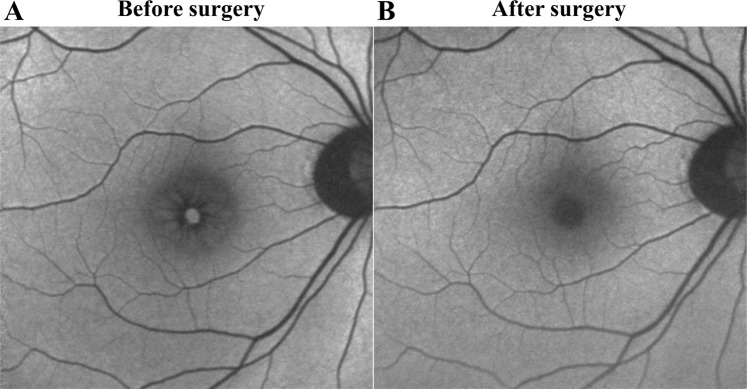
Representative fundus autofluorescence before and 2 weeks after surgery. (**A**) Fundus autofluorescence before surgery in an eye with a macular hole (MH). (**B**) The MH was successfully closed after the surgery and fundus autofluorescence image does not show any hyperfluorescent lines after the surgery.

The swept source-OCT images following the surgery with ILM peeling showed a successful closure of the MH and the displacement of fovea and choroidal intermediate vessels (Fig. 5). Hyperfluorescent lines were not observed in the FAF image in any of the eyes following the surgery (Fig. 6).

## Discussion

Our results showed that the retina was displaced nasally, downwardly, and centripetally toward the center of the optic disc in the macular region. These findings corroborate the findings of an earlier report^10^. The OCT images of the intermediate choroidal slab showed that the choroid was also displaced nasally following the surgery in eyes with an IMH.

The distance between the choroidal bifurcations and the optic disc was significantly decreased in all four quadrants following the surgery. In addition, these distances were positively and significantly correlated with the corresponding preoperative distance between the optic disc and the choroidal bifurcations. These findings indicate that the bifurcations farther from the optic disc had greater displacements following the surgery. In addition, the analyses of the OCT horizontal B scan images showed that the intermediate choroidal vessels were displaced toward the optic disc following the surgery although the degree of the displacement was less than that in the retina.

On the other hand, the angle formed by a line between the center of the optic disc and the retinal or choroidal bifurcation and a horizontal line through the center of the optic disc was not changed following the surgery. In addition, the change in the angle formed by the line from the choroidal bifurcation at week 2 was not significantly correlated with the preoperative angle and was not significantly different in all of the four quadrants. Those results indicate that the choroid was not significantly displaced downwardly following the surgery which differs from the significant downward displacement in the retina.

In the displacement measurements of the choroid using the fovea as the fixed point, the distance between the bifurcations of the choroidal vessel and the fovea was not changed following the surgery. In addition, the amount of displacement of the choroidal bifurcations was not correlated with the preoperative distance between the choroidal bifurcations and the fovea. These results suggest that the choroid was not displaced centripetally following the surgery which differs from the significant displacement of the retina.

Taken together, the choroid was significantly displaced toward the optic disc following the vitrectomy with ILM peeling in eyes with an IMH, but not downwardly, and centripetally. The distance of displacement of the choroid was significantly correlated with that of the retina in each quadrant which suggests that the displacement of the choroid toward the optic disc was corelated to the displacement of the retina.

There have been studies on the retinal displacements in the macular region in eyes following MH closure using ILM peeling with fundus photographs^7–9^, or OCTA en face images^10^. The retinal vasculature is mainly located in the inner retina, meaning that the macular displacement indicates an inner retinal displacement.

There are several reasons why the inner retina is displaced following vitrectomy with ILM peeling. The ILM is the basal membrane of the Müller cells, and it contributes to the integrity of the retina^21^ and less to its elasticity^11,22^. The ILM has about 1,000-fold greater strength mechanically than the other cellular layers, and the ILM comprises 50% of the retinal rigidity^21,23^. In addition, the optic nerve fibers are connected to the lamina cribrosa^24^, and the retinal nerve fibers could move toward the optic disc following a contraction of the nerve fibers. Therefore, the removal of the ILM causes a lack of the structural support of the retina which probably causes the contraction of the nerve fibers where the ILM was peeled and the displacement in the inner retina toward the optic disc.

We were not able to find any studies reporting on the presence of hyperfluorescent lines following the surgery. Shiragami *et al*. reported that hyperfluorescent lines were observed superiorly and in parallel with the retinal vessels in 27 of the 43 eyes (62.8%) following surgery in eyes with a rhegmatogenous retinal detachment (RRD). dell’Omo *et al*. reported that displacements of retina were observed in 44 of 125 (35.2%) eyes following a retinal reattachment in eyes with a RRD^25^. These findings indicated that hyperfluorescent lines can be observed after the inner retinal layer is displaced laterally from the RPE layer as in eyes with a RRD. Our results suggest that the inner retinal layer was not displaced from the RPE layer, and the RPE layer was displaced together with the inner retinal layer following the surgery.

The Müller cells are believed to maintain the structural integrity of the retina as an important role^26^. The sensory retina adheres strongly to the RPE which allows the RPE to transport subretinal fluid at a very high rate against a substantial gradient of hydrostatic or osmotic pressure^27^. This would be related to the observation that the RPE layer was displaced along with the inner retinal layer.

The choroid is comprised mostly of blood vessels histologically and is divided in five layers; Bruch’s membrane, the choriocapillaris, Haller’s layer, Sattler’s layer, and the suprachoroid^28^. The choriocapillaris is approximately 10 µm thickness in the foveal region, and it is a highly anastomosed network of capillaries^28^. The choriocapillaris can be detected by OCTA, but it is difficult to evaluate the displacements of the choriocapillaris layer because its hexagonal and lobular shapes resembles that of the RPE. However, the choriocapillaris layer is presumably not displaced from the RPE layer because the choriocapillaris layer is apposed to the outermost layer of Bruch’s membrane which adheres to the RPE.

OCT *en face* image can detect the vasculature in the choroidal vessels of Haller’s and Sattler’s layer. However, the choroid has the stroma containing extravascular tissue, e.g. elastic fibers and collagen. This means that the choroidal vessels are not adhesively connected in these layers and to the choriocapillaris layer which might cause the gap in the displacement of the choriocapillaris layer and the choroidal vessels in the OCT images. The gap might lead to the differences in the displacements between the retina and choroid following surgery.

Taken together, the peeling of the ILM probably causes the displacement of the inner retina and the degree of displacement would be related to the displacement of the RPE and choroid. However, there is no adhesive connection between the intermediate choroidal vessels causing a gap in the displacement between the choroid and the inner retina following the surgery.

Our result showed that the choroid was not displaced centripetally, i.e., toward the fovea, following the MH closure which was reasonable because if the choroid and the RPE were displaced toward the fovea, then some wrinkling should be observed in the RPE layer over the fovea following the surgery. The distance between the fovea and the bifurcations of the choroidal vessel was in opposite directions between the superior, temporal quadrants and nasal, inferior quadrants. These differences should be related to the fact that the fovea was displaced nasally and inferiorly following the surgery. The displacement of the choroid was less than that of the retina which can cause the distance to be shorter between the fovea and the nasal and inferior bifurcations of the choroid.

There have been many reports about the choroidal thickness and blood flow at the fovea. However, the choroid is displaced following the surgery, and the degree of the displacement in choroid is different from that in retina. This means that the subfoveal choroid following surgery is different from that before surgery, and it can cause differences of the measured area of the subfoveal choroid between before and following surgery. Accordingly, we need to pay attention to the results of the subfoveal choroid, e.g. thickness and blood flow following the vitrectomy with ILM peeling in any kind of retinochoroidal diseases.

This study has several limitations. First, this was a retrospective study on a limited number of participants, which might be the reason why no significant differences were found in the angle and the distance to the fovea in the choroidal displacement. Second, the displacement of choroid was compared with that of retina in each quadrant, and because it is rare that the location of retinal bifurcation is coincident with that of choroidal bifurcation, we did not compare the displacement of retina and choroid directly for each bifurcation. Third, although we confirmed the choroidal displacement in the OCT B-scan images, and we did not measure the distance of displacement using the images because it is difficult to find a fixed point on the image to measure. Fourth, analyses of the 6 × 6 mm^2^ *en face* OCTA image would enable us to analyza a wider field of view. However, the resolution is better with the 3 × 3 mm^2^ *en face* images. Thus, we used the 3 × 3 mm^2^ *en face* images to determine the displacements. Further prospective studies on analyses of the different choroidal vascular layers using a larger number of subjects will be necessary for clarification of the mechanism of the choroidal displacements.

In conclusion, the choroid was displaced toward the optic disc following the vitrectomy with ILM peeling in eyes with a MH. Clinicians need to be aware of this when evaluating the subfoveal choroid following the surgery because the displacement is different between the retina and the choroid.
